# A mechanistic framework for social–ecological mismatches

**DOI:** 10.1093/nsr/nwac130

**Published:** 2022-07-05

**Authors:** Graeme S Cumming

**Affiliations:** ARC Centre of Excellence for Coral Reef Studies, James Cook University, Australia

## Abstract

A minimal social-ecological model, based on the robustness framework, suggests a typology of six different kinds of social-ecological mismatch and a set of general hypotheses about how they might arise.

People rely heavily on experience when interpreting and responding to events in the world around them [1]. As we age, we learn to fit into surrounding landscapes, social groups and rhythms. Like other organisms, our developing brains and bodies are well equipped by our evolutionary history to cope with direct causality and familiar natural phenomena that occur at time frames similar to or shorter than our lifespans and generational times (e.g. vegetation growth, seasonal cycles, animal migrations). However, our focus on particular spatial and temporal scales makes us far worse at identifying and responding to environmental change that is long and slow, occurs over large areas or has complex, indirect causality. Cross-generational exchanges, oral traditions, sacred stories and art, libraries, scientific publications and various other repositories of knowledge retain social memory and learning that help some societies to respond effectively to longer, broader environmental crises [2]. However, in a world in which significant environmental change is affecting more people more rapidly and more extensively than ever before, many past responses are either inadequate or inoperable.

Ecosystems have historically maintained a match between structure, function and process through natural selection. Species that required more resources than a changing landscape could offer went extinct [3]. For example, extinctions of some large frugivorous birds have been attributed to reductions in forest patch size and fruit availability. The seed-dispersing functions of these species, which were no longer matched to the landscape, would have been lost at the same time [4]. By contrast, humans have been able to step outside the immediate constraints of natural selection and reconstruct their niche by developing social and technological solutions to cope with spatial, temporal and functional constraints (e.g. on transport, storage and production of food) [5]. Implementation of these solutions depends heavily on trust and cooperation between people and is regulated through both formal and informal institutions, such as rules, laws, customs and family ties [6]. As the scales of human impacts have increased, people have undoubtedly become better at measuring and thinking about slow, broad-scale change. In the midst of the recent technological explosion of methods and theory, it is easy to forget that fundamental limits on human perception, cognition and social characteristics remain unchanged. Many social–ecological mismatches can be traced back to the limits of human perception, cognition and resulting social processes (e.g. collective action and group vs. individual tradeoffs) that underpin natural resource management.

Traditional approaches to natural resource management have been severely challenged by increases in the geographic scale and intensity of human demands for resources and resulting impacts on ecosystems (‘upscaling’). For example, increases in human population size have resulted in a massive upscaling of both the extent and the intensity of agricultural production over the last century [7]. Upscaling has been driven by growth in the human population; increases in economic production and wealth; increases in inequity; and new technologies, for example in communications, farming and fishing. Different societies have upscaled in different ways, with larger human populations reliant to different degrees on locally or externally produced ecosystem goods and services [8]. Despite the evolution of institutions to prevent over-exploitation and resource degradation, as well as to reduce resource conflicts and limit competition between different resource users, upscaling has set in motion a series of unanticipated global changes that existing governance and management approaches seem incapable of addressing.

Existing frameworks for analysing social–ecological mismatches are descriptive rather than mechanistic; that is, they categorize different kinds of mismatch based on system attributes but do not directly offer explanations for how mismatches arise. Confusion has also arisen over what is explicitly a ‘scale’ mismatch (i.e. relating primarily to the consequences of differences in the relative size, extent, magnitude or speed of interacting elements) and what constitutes another form of mismatch (e.g. relating to differences in the kinds of interaction, expectations or distributions of benefits). For example, inequitable distribution of the proceeds of a community conservation project reflects poor governance and a lack of institutional fit [9], but is not necessarily a problem of scale.

To develop a more mechanistic or process-focused approach that can explain how mismatches arise during upscaling, we need to start with a model of how social–ecological dynamics are structured. Social–ecological systems contain both individual and collective levels of organization as well as geographically structured hierarchies.

There are numerous ways to describe social–ecological systems using models, but many existing approaches are either too general to easily operationalize or too specific for general use [10,11]. From my perspective, one of the best candidates for exploring the mechanisms underlying scale mismatches is the Robustness Framework (also termed the Coupled Infrastructure Systems framework, CIS) [12,13]. It builds on Ostrom's Institutional Analysis and Development framework to operationalize the inclusion of institutions in social–ecological models [12], making it one of the few approaches that directly bridges the gap between social–ecological systems theory, resilience and empirical analysis.

The minimal model of a social–ecological system proposed by the Robustness Framework has four primary components: the ecosystem (resource base); resource users; public infrastructure providers; and public infrastructure. Public infrastructure includes both hard infrastructure (e.g. roads, electricity, water supply) and soft infrastructure (institutions, networks, finance and other socio-economic elements of governance) [13]. Public infrastructure can be gray (built, e.g. roads and bridges); green (ecological, e.g. mangroves and riparian buffers) or blue (e.g. wetlands, rivers) [14]. The Robustness Framework can be applied at a variety of scales, from local to global, recognizing that the relative importance of different elements may change significantly with changes in scale.

Different structural elements described in the Robustness Framework interact through a series of interfaces that can be identified from the intersection of the social–ecological systems and robustness frameworks [6,13] and existing ideas about scale mismatches and institutional fit [9,15]. These frameworks suggest that social–ecological mismatches arise when the system elements on each side of an interface lose cohesion or balance with each other (Fig. 1).

**Figure 1.**
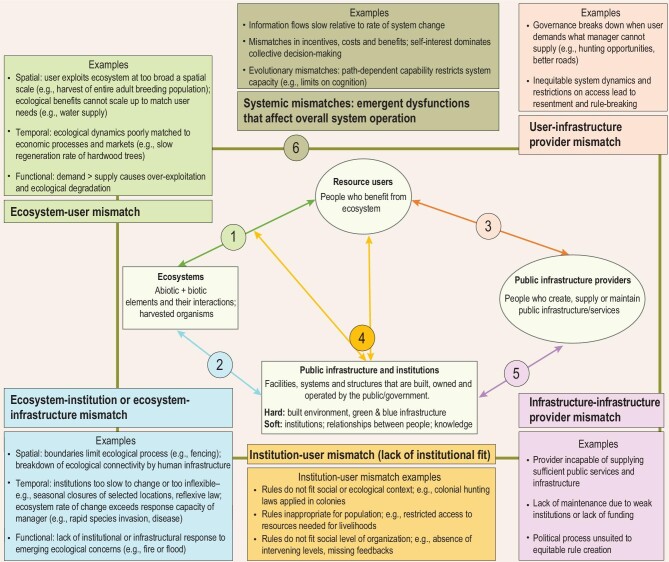
Overview of a causal approach, based on the Robustness (Coupled Infrastructure Systems) Framework, to understanding the different kinds of mismatch that can arise in social–ecological systems. Primarily human elements of the framework are displayed in circles, non-human in rectangles. Mismatches between elements of the social–ecological system arise when components interact, as summarized by the numbered circles (1–6; note that Circle 4 applies to two different arrows, reflecting the direct influence of institutions on people and their indirect influence on interactions between people and nature). Each number describes a different type of mismatch. Five of the mismatch types are connected to specific interactions. The sixth, systemic mismatches, refers to more general, emergent system properties that may cause mismatches at multiple points within the system. Some mismatches that are primarily a consequence of misalignment in social systems can be considered social–ecological because they have indirect (knock-on) effects on ecosystems and are affected indirectly by feedbacks from ecosystems. For example, fencing of wildlife areas may cause tension between resource users and infrastructure providers (Circle 3) that then leads resource users to poach wildlife from ecosystems (Circle 1).

If increasing human demand, inequity and our ever-increasing technological capacity to manipulate and exploit ecosystems are the root causes of social–ecological mismatches, what are the solutions? In addition to reducing demand and improving equity, one proposed governance solution is the development of scale-sensitive, polycentric governance that uses local knowledge and environmental monitoring to guide collective action across different scales [16]. Polycentric governance incorporates many centers of decision-making, often at different hierarchical levels or geographic scales, which are formally independent of each other but functionally interdependent [17]. A polycentric governance system could in theory base global governance on local knowledge and manageable levels of complexity while contributing to addressing cross-scale environmental problems, potentially providing the coordination across boundaries and systems that is needed to resolve social–ecological mismatches [18]. However, efforts to deliberately design scale-sensitive governance approaches still lack critical foundational knowledge that is needed to achieve an effective realignment of social and ecological processes at each of the interfaces defined in Fig. 1.

Among the critical gaps in our understanding of the processes that generate and resolve social–ecological mismatches, three in particular stand out. First, we have a poor understanding of the relative importance of endogenous and exogenous drivers of change and how they change with scale. Societies and their institutions for natural resource management must balance endogenous pressures (e.g. demand for resources and economic growth) with exogenous constraints (e.g. limitations on capacity of ecosystems to meet demand; broader political pressures) at different scales and levels of organization. Achieving this balance requires the maintenance of feedbacks between key system elements (e.g. populations of harvested food species and harvesting allocations), while defending common pool resources from the extremes of either a free-for-all situation or capture by a wealthy elite.

Second, few studies have both addressed and controlled for scale-crossing behaviors and mechanisms (both social and ecological) and the ways in which structural change and the formation of networks can either cause or resolve social–ecological scale mismatches. Global economic networks of supply and demand, for example, connect local communities around the world. These networks can enhance resource provisioning across the network in the face of local disruptions (e.g. regional drought or conflict) but they can also severely reduce awareness of localized environmental impacts, creating a social–ecological mismatch [19]. Changes in governance are often structural (e.g. introducing new institutions or management structures), but their corresponding functional outcomes (e.g. impacts on the environment) are poorly understood and may be counter-intuitive, particularly if they unexpectedly change individual perceptions of costs and benefits in public goods problems. In Australia, for instance, changes in land-clearing laws through the introduction of the Vegetation Management Act in 1999 may have unintentionally caused a wave of deforestation [20].

Third, we lack rigorous theories of transformation and collapse. Efforts to resolve social–ecological mismatches are often *ad hoc* experiments, with varying levels of success. The development of a theoretical framework that guides transformational interventions will be critical for future efforts to achieve sustainability and learn from past experience. The beginnings of a theory of both positive and negative transformational change already exist in the published literature [21,22], but these ideas still require formalization and operationalization into more rigorous, testable hypotheses that can be confronted with empirical evidence.

Overall, and speaking subjectively, research on social–ecological-scale mismatches appears to be becoming increasingly quantitative and mechanism-focused. This promising trend must now be coupled to theoretical frameworks that guide and direct our understanding of how transformational change, scale-crossing actions and balancing endogenous and exogenous pressures can contribute to enhanced social–ecological resilience and sustainability.
